# Cytotoxic or Not? Disclosing the Toxic Nature of Carbonaceous Nanomaterials through Nano–Bio Interactions

**DOI:** 10.3390/ma13092060

**Published:** 2020-04-29

**Authors:** Joanna Czarnecka, Marek Wiśniewski, Natalia Forbot, Paulina Bolibok, Artur P. Terzyk, Katarzyna Roszek

**Affiliations:** 1 Department of Biochemistry, Faculty of Biological and Veterinary Sciences, Nicolaus Copernicus University in Toruń, Lwowska 1, 87-100 Toruń, Poland; j_czar@umk.pl (J.C.); n_forbot@umk.pl (N.F.); 2 Physicochemistry of Carbon Materials Research Group, Faculty of Chemistry, Nicolaus Copernicus University in Toruń, Gagarina 7, 87-100 Toruń, Poland; marekw@umk.pl (M.W.); pbolibok@umk.pl (P.B.); aterzyk@chem.umk.pl (A.P.T.)

**Keywords:** carbonaceous nanomaterials, carbon nanotubes, nano–bio interactions, protein corona, cytotoxicity, long-term exposure, mesenchymal stem cells

## Abstract

The cytotoxic influence of two different carbonaceous nanomaterials on human mesenchymal stem cells (MSCs) cultured in vitro was compared in the short (1–3 days) and long term (up to 60 days). Amorphous carbon and single-walled carbon nanotubes were chosen and evaluated due to their contrasting physicochemical properties. Both materials, though supposed similarly low-toxic in basic short-term cytotoxicity assays, demonstrated dramatically different properties in the long-term study. The surface chemistry and biomolecule-adsorption capacity turned out to be crucial factors influencing cytotoxicity. We proved that amorphous carbon is able to weakly bind a low-affinity protein coat (so-called soft corona), while carbon nanotubes behaved oppositely. Obtained results from zeta-potential and adsorption measurements for both nanomaterials confirmed that a hard protein corona was present on the single-walled carbon-nanotube surface that aggravated their cytotoxic influence. The long-term exposure of the mesenchymal stem cells to carbon nanotubes, coated by the strongly bound proteins, showed a significant decrease in cell-growth rate, followed by cell senescence and death. These results are of great importance in the light of increasing nanomaterial applications in biomedicine and cell-based therapies. Our better understanding of the puzzling cytotoxicity of carbonaceous nanomaterials, reflecting their surface chemistry and interactions, is helpful in adjusting their properties when tailored for specific applications.

## 1. Introduction

Carbonaceous nanomaterials (CNMs), including carbon nanotubes, graphene oxide, carbon black, fullerenes, nanodiamonds, and other nanostructures, belong to a rapidly growing family of advanced materials that integrate the distinctive properties of sp^2^-hybridized carbon bonds with unique physicochemical properties at the nanoscale. Each member of the CNM family exhibits inimitable features, e.g., a high surface-to-volume ratio, mechanical strength, acid and base resistivity, and electrical conductivity. Their widespread application in many industrial areas, including energy storage, electronics, sensors, agriculture, and the cosmetic and pharmaceutical industries [1,2,3,4], is accompanied by increased human exposure. Carbon-based nanomaterials are also promising candidates for various applications in medical fields such as diagnostics, therapy, drug delivery, and imaging [5,6], and in cell-based approaches, including stem-cell culture and differentiation [7,8]. However, engineered CNMs such as carbon nanotubes and spherical amorphous nanomaterials, due to their similarity to combustion-derived nanoparticles, may cause adverse effects on human health via the accumulation of entire particles or their degradation products [9]. Thus, their cytotoxicity should be carefully assessed to increase prediction of regarding nanomaterial fate and safety in humans.

At the start of researching and developing any carbonaceous nanomaterial, it is necessary to evaluate its potential toxic effects, and identify the physicochemical properties that are responsible for toxicity. Additionally, it is crucial to clarify the molecular mechanisms of carbon nanomaterial-induced cytotoxicity [3]. Nanotoxicology has received much scientific attention in recent years, but published results have not led to any consensus on the toxicity profile of pristine or functionalized CNMs. To in vitro analyze a short-term effect of any material, different cytotoxicity assays are used in mammalian cell lines as models. Upon exposure to nanoscale materials, animal and human cells may be acutely damaged, and their functionality may be perturbed through different modes of action, often depending on material physicochemical properties underpinning surface interaction or internalization abilities [10,11,12,13].

Carbon nanotubes are categorized into two types: single-walled carbon nanotubes (SWCNTs) and multi-walled carbon nanotubes (MWCNTs), which seem to be the most frequent material for cytotoxic tests on various cell lines. Due to their poor solubility and potential toxicity, they are commonly modified through the functionalization of the CNT surface [13]. Despite their different surface properties, increased production of reactive oxygen species (ROS) is one of the most common toxicity mechanisms of CNTs. It triggers oxidative stress, inflammation, and lipid peroxidation, and results in damage to proteins, the cell membrane, and DNA [9,14]. It was also observed that different types of SWCNT can induce the deterioration of cell growth through the adsorptive interaction between SWCNT and components of the medium [15], or affect the adhesive ability of cells [1].

On the other hand, amorphous carbon in its different forms seems to be cytocompatible, as no toxic influence was reported even at high concentrations (exceeding 1.0 mg/mL) [16,17]. Amorphous carbon deposited on stainless-steel substrates supports the in vitro growth and biomineralization of human osteoblasts, and favors osteoinduction in rat bone-marrow-derived mesenchymal stem cells [16]. According to Popov et al. [18], amorphous carbon films were nontoxic for osteoblast-like cells and pneumocytes. Exposure of the films to simulated body fluid confirmed that they are bioinert, so this material has been postulated as suitable for implants and other biomedical applications. Amorphous carbon nanomaterial was even suggested as a beneficial biomaterial substrate for supporting the cell adhesion and proliferation of neuronal cells in the context of their applications as artificial nerve implants. However, selected parameters of cell viability were determined after 4 days in culture, and no long-term exposure was evaluated [17].

To date, the investigation of the cytotoxic properties of CNMs has been confounding and inconclusive since opposing results have been reported throughout the literature, even for closely related materials. Divergent outcomes might be related to, for example, the subtle differences in the physicochemical properties or structure of carbon nanomaterials, the types of cells engaged in the tests, methods of particles dispersion, and used cytotoxicity tests [13,19]. Despite progress in analytical techniques and protocols, assessing the cytotoxicity of carbon nanomaterials still poses considerable challenges. One of the major concerns about CNM toxicity determination is the lack of standards referring to the dose, exposure time, and measurable parameters of cytotoxicity. The development of reliable toxicity-screening tools is of crucial importance for the near future [19].

Attempts to understand how nanomaterial characteristics and properties may encompass increased toxicological effects for human tissue and cells require the use of harmonized, multiendpoint protocols, and careful in vitro assessment [3]. It is not yet fully understood which properties of carbon nanomaterials, e.g., surface area, mass concentration, surface chemistry, particle geometry, dispersibility, metal impurities, a combination of these features, or some other factors, play a central role in cytotoxicity [1,3]. One of the factors influencing nanomaterial toxicity is their surface structure and chemistry that defines, inter alia, the material ability to interact with biomolecules. Biointeractions include interactions with proteins that are abundant in the extracellular environment and inside the cell, and other biomolecules like nucleic acids or lipids. The structure and composition of the protein coating is different on various surface chemistries, but can also be complex and dynamic [20,21].

For better understanding nano–bio interactions, studies have made great effort to reveal unknown mechanisms in both in vitro and in vivo studies [1,13]. Interactions with proteins leading to the creation of the so-called protein corona are often noted as the method to alleviate nanomaterial toxicity, make them biocompatible with the human body, and beneficial for therapeutic use [3,20,21]. On the other hand, protein-corona formation may also induce undesirable and sometimes opposite results: increasing or inhibiting cellular uptake, hindering active targeting, and mitigating or aggravating cytotoxicity [22]. Thus, creating specific protein coronas as a sort of natural nanomaterial functionalization, as well as the potential impact of biocorona on the fate of nanomaterials in the cells and tissue, should not be ignored [2,8,21,23]. There are still several challenges to overcome in this field, e.g., issues concerning the composition of protein corona, and the ability of protein-coat components to interact with living cells or to play a crucial role in determining cell fate [7,21].

The successful translation of nanomaterials for biomedical applications should be based not only on a detailed understanding of their physicochemical properties, but also on revealing the biological interactions of these materials and mechanisms that govern their uptake and biodistribution [3]. In the presented work, we effectively combined cytotoxicity assessment in human mesenchymal stem cells after their long-term exposure to CNMs with attempts to reveal the mechanism of CNM-mediated influence based on their surface chemistry and interactions with biomolecules. To the best of our knowledge, any previously published data combined the short- and long-term effects of CNM toxicity with their biointeraction capability in mesenchymal stem cells cultured in vitro. The presented study focuses on a new approach to CNM toxicity, disclosing the cytotoxic properties of CNMs through their ability to interact with proteins abundant in the extracellular matrix. The complete understanding of nano–bio interactions is the solution to the puzzling issue of carbonaceous-nanomaterial toxicity, reflecting their surface chemistry. It also paves the way for adjusting CNM properties tailored for specific applications.

## 2. Results

**Figure 1:**
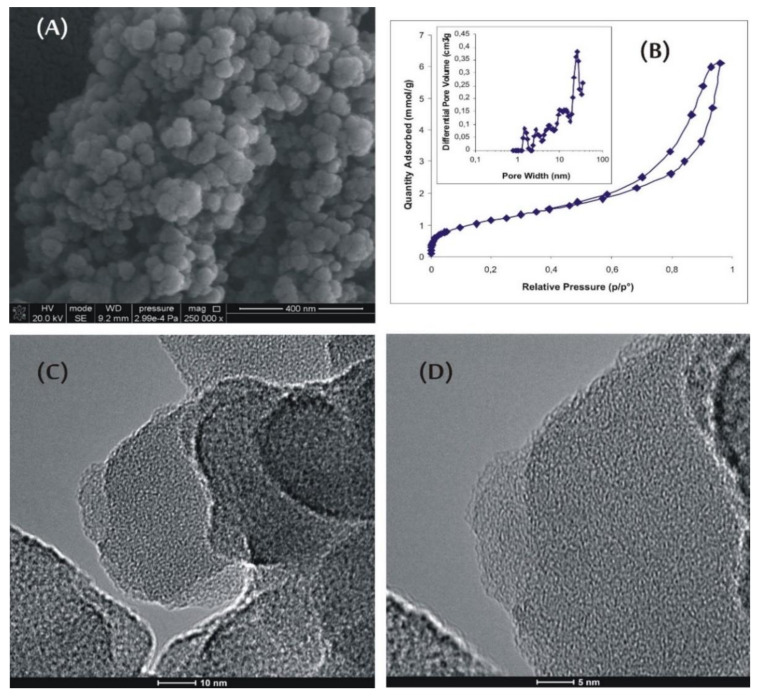
Morphology of amorphous-carbon-nanomaterial (ACNM) sample: (**A**) SEM image, (**B**) low-temperature (77 K) N_2_ adsorption isotherm, and (**C**,**D**) HRTEM images.

Typical scanning electron microscopy (SEM) and high-resolution transmission microscopy (HRTEM) images of amorphous-carbon-nanomaterial (ACNM) samples are shown in Figure 1. The HRTEM images show the spherical shape of nanoparticles having a very rough surface (Figure 1C,D). The observed roughness was due to small aggregating spheres forming primary structures, shown in Figure 1A (with a diameter of about 30 nm).

**Figure 2:**
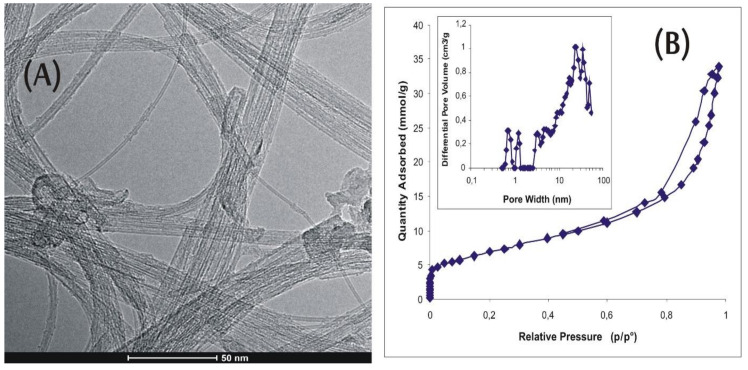
Morphology of single-walled-carbon-nanotube (SWCNT) sample: (**A**) HRTEM image; (**B**) low-temperature (77 K) N_2_ adsorption isotherm.

The primary spheres seemed to be rather ultramicroporous and highly amorphous. It is clear that they formed the secondary pore structure with a pore diameter up to 35 nm. Additionally, long and partially opened, commercially available, single-walled carbon nanotubes (SWCNT; Figure 2A) were studied for comparison. The tubes possessed a very small number of side-wall defects and a small amount of amorphous carbon (Figure 2A,B).

These CNM specimens were chosen due to the fundamental differences in their properties (Table 1). While SWCNT samples are tube-shaped, highly graphitized, with low oxygen content, and a small number of functional groups, the ACNM materials are spherical in shape, with lower surface area, higher oxygen content, and much higher number of functional groups on the surface.

**Figure 3:**
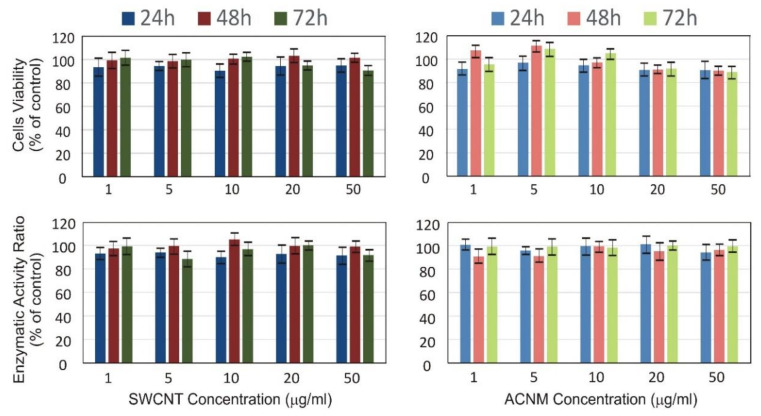
(**top**) Cell viability and (**bottom**) membrane damage of mesenchymal stem cells exposed to (**left**) SWCNT and (**right**) ACNM in short-term evaluation. Bars represent mean ± SD.

Figure 3 presents results from a standard, i.e., short-term in vitro cytotoxicity evaluation using human mesenchymal stem cells exposed to concentrations of CNMs ranging from 1 to 50 µg/mL for 24, 48, and 72 h. Our results from the MTT (3-(4,5-Dimethyl-2-thiazolyl)-2,5-diphenyl-2H-tetrazolium bromide) assay showed that MSCs during short-term exposure to different SWCNT and ACNM concentrations slightly decreased their viability after 24 h incubation, whereas a longer culture resulted in compensating the number of cells, at least for concentrations not exceeding 10 µg/mL (Figure 3). Low (similar to control cells) and stable lactate dehydrogenase (LDH) activity in all nanomaterial concentrations and during incubation time of up to 72 h suggests that the disintegration of the cell membranes was not significantly higher than that in control cells.

**Figure 4:**
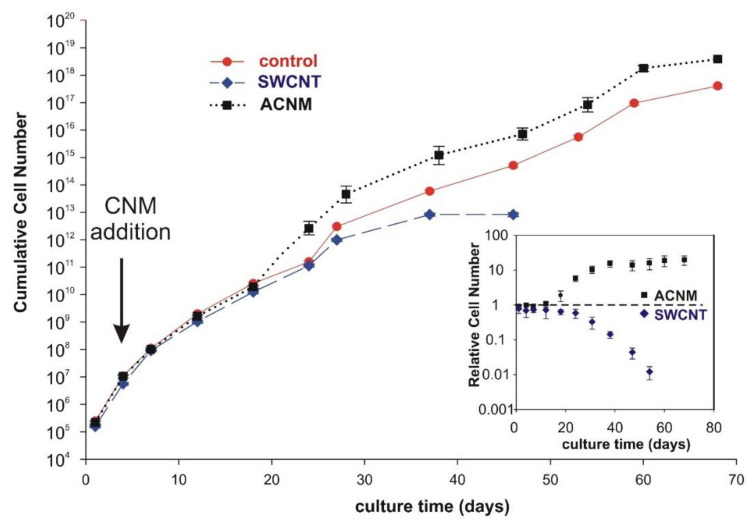
Growth curve of mesenchymal stem cells during long-term evaluation of tested materials. Inset presents cell number relative to control. Bars represent mean ± SD.

**Figure 5:**
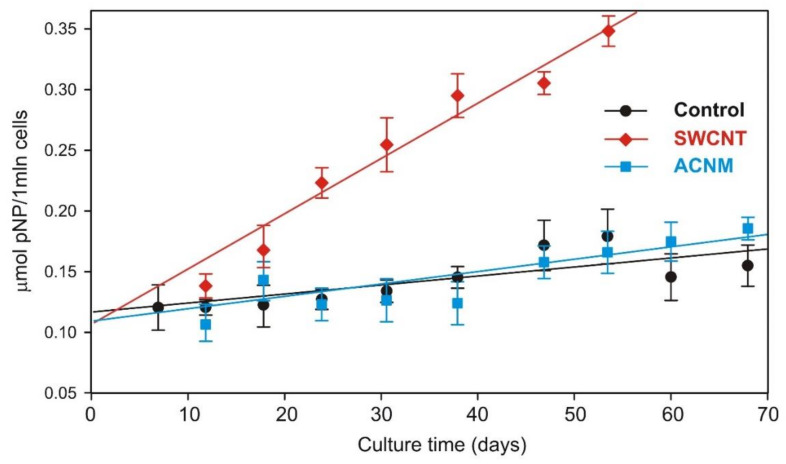
Senescence-associated β-galactosidase activity assay during long-term exposure of mesenchymal stem cells. Bars represent mean ± SD.

Data from the long-term exposure of MSC cells to carbonaceous materials in a concentration of 10 µg/mL showed that there were no considerable differences in the growth rate of MSCs up to approximately 20 days in culture (Figure 4). However, longer exposure of cells (>20 days) caused changes in their growth rate. Cells cultured in the presence of SWCNT showed decreasing cell proliferation, whereas cells exposed to ACNM proliferated faster than the control cells. This resulted in a higher by one order of magnitude cumulative number of cells at subsequent culture stages. Data from Figure 5 show the β-galactosidase activity of mesenchymal stem cells at pH 6.0, which is a good reflection of the senescence process. Senescence-associated β-galactosidase activity was clearly increased only in the presence of the SWCNT samples.

**Figure 6:**
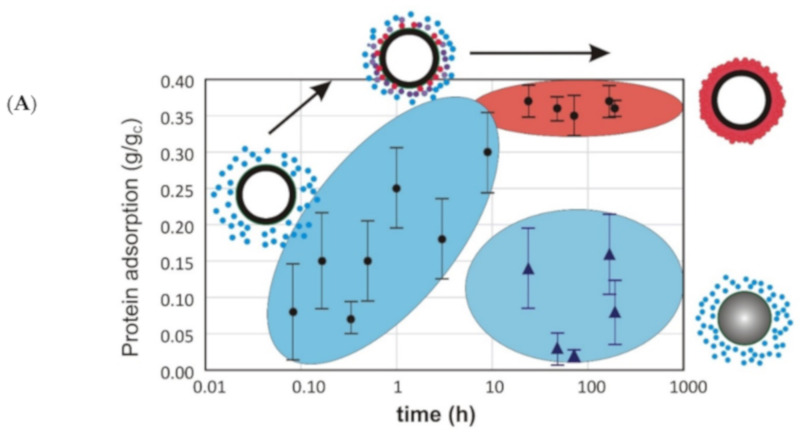

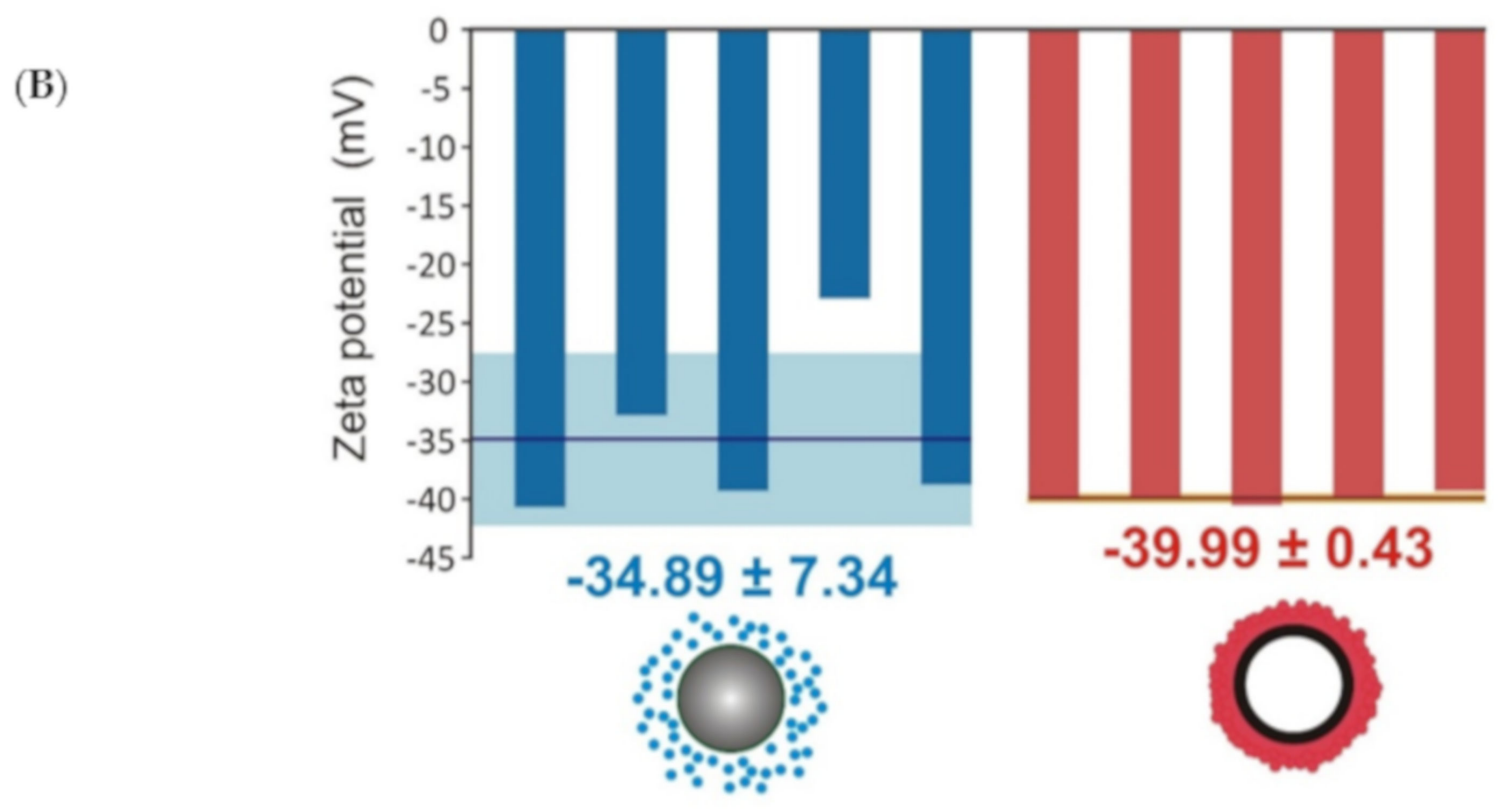
Protein-adsorption (**A**) and zeta-potential measurements (**B**) of carbonaceous nanomaterials: single-walled carbon nanotubes (empty circles) and amorphous carbon (filled circles).

**Figure 7:**
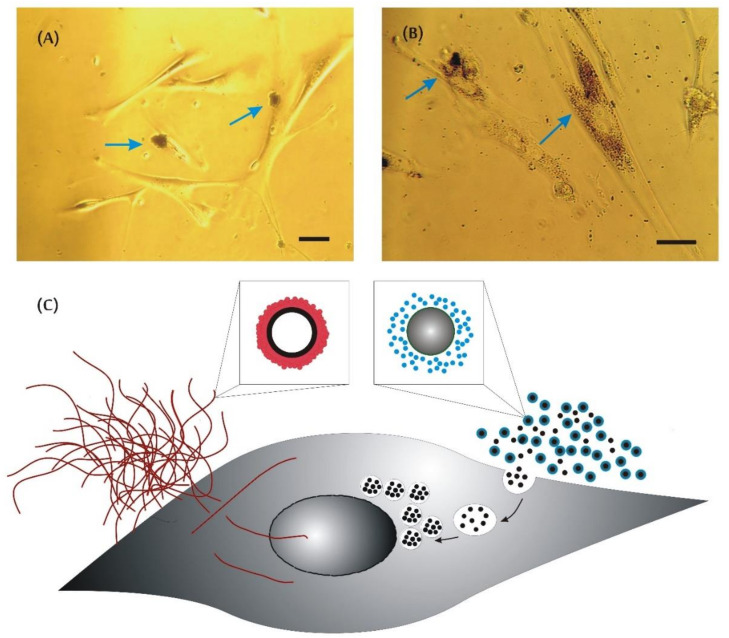
Mesenchymal stem cells after 30 days of culture in presence of (**A**) SWCNT and (**B**) ACNM. Bars indicate 10 µm; arrows show CNM agglomerates (**A**) outside or (**B**) inside cells. (**C**) Proposed mechanism of carbonaceous-material interaction with proteins and mesenchymal stem cells.

To shed more light on the phenomena of the different influence of CNMs, we assessed the protein-adsorption rate and zeta potential of the tested carbonaceous nanomaterials (Figure 6). Adsorption studies revealed that carbon nanotubes adsorb a steady amount of proteins on their surface, whereas the protein corona on the amorphous carbon specimen was negligible. This is in good agreement with the microscopic data – SWCNTs were larger and could not be internalized by the cell, probably due to the protein coat and/or aggregation (Figure 7A), while ACNMs easily penetrated the cells (Figure 7B). The proposed conception of the carbonaceous-material interaction with proteins and mesenchymal stem cells is presented in Figure 7C.

## 3. Discussion

Among different types of nanomaterials, CNMs belong to the most promising, offering extensive potential applications. Much progress has been made in evaluating carbonaceous-nanomaterial toxicity both during in vivo and in vitro studies, but these evaluations often remain confounding and incomplete [11,23,24,25]. Although in vivo results are valuable, the involvement of experiment animals should be limited in accordance to current European Union (EU) directives [26]. With this in mind, careful and comprehensive approaches to in vitro cytotoxicity assessment are often called for.

Carbon nanotubes are a widely studied nanomaterial, but some in vitro toxicity tests showed that nanotubes are nontoxic, especially at low concentrations [27,28,29], whereas some researchers claimed that they could be more toxic than asbestos [9,27,30]. What is the cause of these apparently double-faced toxic properties of carbon nanomaterials? The nanomaterial-toxicity issue is a significant and complex concern, and as most of the studies in this area are not using the same methodologies and standards, so a comparison of the results is difficult. Moreover, published data indicate many possible mechanisms of CNM-mediated cell damage that should be concerned. In our opinion, the study of CNM toxicity is still very recent, and existing toxicological data are fragmentary; therefore, the multiendpoint evaluation of nanomaterial toxicity is not to be underestimated.

Chemical analyses showed that many physicochemical CNM properties, e.g., purity, shape, length, diameter, and aggregation ability may affect sample toxicity [1,31]. Therefore, we chose and compared single-walled carbon nanotubes representing graphitized carbons, and amorphous material with morphology typical for hydrothermally prepared samples [32,33]. Despite differences in the structure of the analyzed nanomaterials, literature data described both of them as biocompatible and nonimmunogenic [27,28,29]. In addition, both nanomaterials offer a new perspective of their application as nanovehicles in drug-delivery systems [34,35]. Recently, oxidized carbon particles were described, prepared from graphite as a starting material, with the outstanding ability to penetrate a lipid bilayer membrane [36]. These particles were recommended as offering great possibilities in drug-delivery strategies. Regrettably, their biocompatibility was tested in vitro for only 24 h. The results presented in our study indicate that apparently nontoxic materials proved to exhibit dramatically different properties in long-term exposure.

We demonstrated CNM influence on cytophysiology after short- and long-term exposure of human mesenchymal stem cells. The standard cytotoxicity evaluation performed in a concentration- and time-dependent manner (up to 72 h of in vitro culture) did not reveal drastic differences in the toxicity of the tested specimens, which is completely in line with previously published results, e.g., by Mihalchik et al. [37] or Figarol et al. [38]. Cell viability at low concentrations (below 10 µg/mL) of SWCNT and ACNM increased slightly, predominantly due to an increased proliferation rate (that was observable after 72 h of culture). The decrease in cell viability at the highest concentration of 50 µg/mL was expressed slightly more with ACNM than with SWCNT. Surprisingly, cell-membrane damage was inconsiderable after MSC exposure to amorphous carbon particles.

Prolonged experiments allowed to distinguish the cytotoxic properties of ACNM and SWCNT. Results from the growth curve revealed that the amorphous carbon was rather biocompatible after long-term exposure, while carbon nanotubes were not. A repeated SWCNT dose of 10 µg/mL (far below EC_50_ as calculated from short-term exposure) initiated the toxic effects, leading to a decrease in cell-proliferation rate, and faster senescence and cell death. The senescence process is one of the reasons limiting MSC “stemness” and biological potential, and increased β-galactosidase activity at pH 6.0 is a good reflection of the senescence process in MSCs exposed to the repeated dose of SWCNT.

Undoubtedly, protein coatings modify and control the behavior of nanoparticles, potentially altering their aggregation state and uptake by cells, and cellular response, which, in turn, may influence their fate and hazard to human health [22]. Protein adsorption also underlies the depletion of the culture medium in biologically active proteins, including hormones, growth factors, and enzymes. Thus, we thoroughly examined the physicochemical properties of both nanomaterial specimens and linked them with toxicological data. The large surface area of SWCNT suggested the material ability to adsorb different biomolecules, while higher oxygen content and much higher number of functional groups on the ACNM surface could favor nano–bio interactions. Protein adsorption on the SWCNT surface turned out to be significantly higher than that on ACNM, and stable over the time. The zeta potential measured in a time-dependent manner reflects the stability of the protein corona as suggested by the literature data [39]. The hard corona comprised strongly bound proteins, while the soft corona was dynamic and was formed from loosely bound proteins that stayed in a dynamic equilibrium with the biological microenvironment [21,22]. The zeta-potential values obtained for the studied CNMs indicated that a hard protein corona was present on the SWCNT surface, whereas a soft and labile protein coat was loosely adsorbed on the ACNM surface.

The possible mechanisms of the studied nanomaterials’ influence on cell growth were underpinned with the kind of formed protein coronas. In short-term exposition, a protein corona coating the nanomaterial samples (whether strongly or weakly interacting) seemed to play a protective role in the cell culture. The toxicity of the protein-coated materials decreased, which was consistent with the literature data. Only recently, it was reported that the cytotoxicity of carbon nanotubes and reactive oxygen species formation in vitro were reduced for serum albumin–antioxidant–CNT complexes relative to those of serum–CNTs or CNTs alone [14]. Long-term cell exposure, however, is associated with a gradual increase in nanomaterial–protein interaction and impaired protein composition in the extracellular environment. Nanotubes are not able to release proteins from their hard corona, so surface-bound regulatory proteins are not available for rapidly proliferating cells. On the other hand, the labile protein coat on amorphous carbon, which was in equilibrium with the environment, was able to release proteins. The low toxicity of “pure” material and its low reactivity do not cause negative effects inside and outside the cell, which makes ACNM biocompatible and nontoxic for cells in long-term exposure.

Additionally, from microscopic data, one can conclude that SWCNT aggregates are large and predominantly visible on the MSC surface (Figure 7A). They seem to not be internalized by cells, and as a result, are able to interact with surface receptors/external enzyme domains/channels, or decrease the adhesion ability. According to Wick et al. [40], the toxic effect of highly agglomerated CNTs was more pronounced, and it stayed in good agreement with our present results. However, according to recent reviews, CNTs can easily cross a variety of biological barriers and pass through the membrane into the cytoplasm [23,41]. On the basis of our previous reports [19,34,35], it can also be assumed that single tubes are able to penetrate cells, and the aggregation state is one of the essential factors in determining the toxic potential of CNTs. Thus, toxic effects observed at the cellular level, i.e., growth deterioration or faster cell senescence, could both result from small membrane damage or be exerted from the inside of the cell (Figure 7C). On the other hand, the ACNM material easily penetrated MSCs and accumulated in the cytosol (Figure 7B), presumably via the endocytosis pathway, as suggested by some researchers [23,36]. The endocytosed material remained partially coated with the vesicle membrane and did not cross the nuclear-envelope barrier. Even if it did, it could not interact with regulatory proteins, growth factors, or DNA due to its low adsorption capacity. Additionally, it has previously been observed that bare silica nanoparticles exposed to cells have stronger adhesion to the cell membrane and higher internalization efficiency, in comparison to what is noticed when a preformed biocorona is present on the nanoparticle surface [42]. One can conclude that the aggregation rate, together with surface size and properties, as well as protein coronation are the most essential factors influencing each other and resulting in specific nanomaterial cytotoxicity. However, this issue still requires more detailed studies.

Taken together, surface chemistry and biomolecule adsorption turned out to be crucial properties influencing carbonaceous-material cytotoxicity. From both studied specimens, only amorphous carbon was characterized by low adsorption capability and cytocompatibility, even when applied in the repeated dose and despite internalization ability. Such material seems to be an excellent carrier for novel therapeutic applications based on drug delivery directly into the cell.

## 4. Conclusions

One of the major challenges in the development of nanomedicines is the choice of the right material, which notably determines subsequent biological responses. Despite considerable efforts in the field of nanotechnology, fewer nanoparticles and nanomaterials than expected have been applied in clinical trials. The wide gap between efforts and effective clinical translation is, at least in part, due to multiple factors governing both in vitro and in vivo environments, a poor understanding of the nano–bio interface, and misinterpretation of data collected in vitro, all of which reduce prediction accuracy regarding nanomaterial fate and safety in humans. The thorough analysis of CNM surface chemistry and nano–bio interactions is mentioned in the very recent literature. Hence, there are still several challenges to overcome in this field, e.g., issues concerning the composition of protein corona or the ability of these components to control cell cytophysiology.

Our studies here revealed that carbon nanotubes adsorb a remarkable amount of proteins on their surface, whereas amorphous carbon does not. Surprisingly, protein coronation aggravates the toxic nanomaterial influence on cell physiology. The long-term exposure of mesenchymal stem cells to SWCNT, coated by strongly bound proteins, showed a significant decrease in cell-growth rate, followed by cell senescence and death. Amorphous carbon, with a soft and labile protein coat, seemed to still be biocompatible.

We anticipate that these data are helpful in establishing a new approach to CNM cytotoxicity testing that considers nano–bio interactions. Differences in nanomaterials’ surface chemistry, leading to different interactions with proteins, can be harnessed in designing tailored properties of engineered nanomaterials for biomedicine.

## 5. Materials and Methods

### 5.1. Carbonaceous-Nanomaterial Preparation

Commercially available, high-purity, opened single-walled CNTs (purchased from NanoAmor, Houston, TX, USA) were used in experiment studies and termed as SWCNTs. The detailed characteristics of SWCNTs (microscopic characteristic, the values of specific Brunauer–Emmett–Teller (BET) surface area, the enthalpy values of immersion in benzene and methanol, the concentration of surface acidic and basic groups, and the pH of suspension values) were previously given by Werengowska-Ciećwierz et al. [34].

Another newly synthesized material in our lab used in the study can be classified as amorphous CNM, and it is denoted as ACNM. A single-step process of hydrothermal synthesis was carried out in a Parr reactor (6 h, 473 K, 20 MPa) from 2 wt % chitosan solution in 2% acetic acid that allowed for the formation of nanospherical carbons with a diameter of about 30 nm (see Figure 1). Low temperature (77 K) N_2_ adsorption, SEM, HRTEM, and pH of the suspension measurements were used to examine the properties of the new material. Adsorptive and acid–base properties, which are crucial for interaction with the cell and extracellular-matrix components, were also evaluated.

The tested carbon materials were dispersed by ultrasonication (3 h) in a concentration of 1 mg/mL in 10 mmol/L sterile phosphate-buffered saline (PBS), pH = 7.4. The appropriate dose of the solution was added to the cell-culture medium to obtain final concentrations of 1, 5, 10, and 50 μg/mL.

### 5.2. In Vitro Cell Culture

Human umbilical-cord mesenchymal stem cells (MSCs) were purchased from PromoCell (Heidelberg, Germany). Cells were grown in Mesenchymal Stem Cell Growth Medium^®^ with 10% Supplement Mix^®^ (both from PromoCell) in a CO_2_ incubator with 5% CO_2_. Approximately 5 × 10^5^ cells were seeded in each well of a 12-well plate for 24 h.

For the short-term experiments, CNM samples were added to the growing MSCs in the concentration range of 1–50 μg/mL (in 1 mL volume of growth medium) and incubated for 24–72 h without media refreshment. In the long-term exposure, CNM samples were added to the growing MSCs in a concentration of 10 μg/mL, and cultured at least for 7 passages (circa 40 days) for SWCNT and 10 passages (circa 60 days) in the case of the ACNM material. The culture medium with CNM of 10 μg/mL concentration was changed every 3 days. After reaching the subconfluency state, the cells were subcultured and again exposed to the tested materials in appropriate concentrations.

### 5.3. Viability Assays

Trypan blue staining and MTT assays for cell viability, as well as the lactate dehydrogenase (LDH) activity test for cell-membrane damage, were performed in triplicate for cells after short- and long-time exposure to CNMs. A detailed description of the cytotoxicity tests was previously given by Werengowska-Ciećwierz et al. [19]. Briefly, the MTT assay was conducted as follows: MTT solution in a concentration of 1 mg/mL was added to each well, with cells exposed to different concentrations of the nanomaterials. After 30 min of incubation, the solution was discarded, and formazan crystals were dissolved in dimethyl sulfoxide (DMSO). Absorbance was spectrophotometrically measured at 570 nm (using nontreated cells as control).

LDH activity was determined in the culture medium by measuring the decrease in NADH (β-nicotinamide adenine dinucleotide, reduced disodium salt). The decrease in the amount of NADH was directly correlated to the increase in the number of damaged cells. For measuring LDH activity, the culture medium was collected from the wells with MSCs exposed to different nanomaterial concentrations. The LDH activity test was performed as follows: 100 μL of NADH (1.25 mg/mL) and 100 μL of sodium pyruvate (2.5 mg/mL) were added to 600 μL of the culture medium. Absorbance at 340 nm was spectrophotometrically measured. The number of damaged cells was compared to the untreated control sample, also considering the positive control treated with 1% Triton X-100 as 100% damaged cells.

### 5.4. Functional Assay-Senescence-Associated β-Galactosidase Activity

β-galactosidase activity at pH 6.0 was determined to evaluate cellular senescence. The untreated cells (control) and cells exposed to prolonged treatment with carbonaceous nanomaterials were collected at subsequent passages, counted, and then lysed using a lysis buffer with 1% Triton X-100. Aliquots of 0.1 mL cell lysates were added to 0.2 mL p-nitrophenyl-β-D-galactopyranoside (NPG) in a concentration of 4 mg/mL in 0.25 M phosphate buffer, pH = 6.0, containing 1 mM MgCl_2_ and 45 mM β-mercaptoethanol. After 120 min of incubation, the enzymatic reaction was stopped by the addition of 0.5 mL 0.1M NaOH. Absorbance of the released p-nitrophenol was measured at λ = 405 nm.

### 5.5. Adsorption Studies

The tested carbon materials were dispersed by ultrasonication, and suspended in concentration of 600 μg/mL in 0.05 mM sterile PBS buffer with 1 % fetal bovine serum (FBS, about 600 μg/mL of total protein). After a specified period of incubation (from 5 min to 192 h) at 4 °C, suspension was centrifuged at 9200 g for 10 min, and protein concentration was spectrophotometrically assayed at λ = 280 nm. The remaining carbon nanomaterials were used for zeta-potential measurements.

### 5.6. Zeta Potential Measurements

Zeta potential was measured using Nano Plus HD (Micromeritics, Norcross, GA, USA) at 25 °C. All samples were suspended in 0.05 mM PBS buffer, pH = 7.4, to obtain a concentration of 10 μg/mL.

### 5.7. Statistical Analysis

All experiments were repeated at least three times, and qualitatively similar results were obtained. Experiment data are expressed as mean ± SD of the triplicate independent samples. The presented photographs are representative for the three tested series of experiments.

## Figures and Tables

**Table 1:** Basic physicochemical characteristics of studied samples.

Sample	BET Surface Area (m^2^/g)	Suspension pH	Oxygen Content (%)	Acidic Groups Ca (mmol/g) (mmol/m^2^)	Basic Groups Cb (mmol/g) (mmol/m^2^)	Ca/Cb
SWCNT	570	6.78	5.86	0.1901 (3.33 × 10^−4^)	0.0555 (1.00 × 10^−4^)	3.43
ACNM	100	6.64	26.15	0.5787 (5.79 × 10^−3^)	0.4981 (4.98 × 10^−3^)	1.16

## References

[1] Liu Y., Zhao Y., Sun B., Chen C. (2013). Understanding the toxicity of carbon nanotubes. Acc. Chem. Res..

[2] Louro H. (2018). Relevance of Physicochemical Characterization of Nanomaterials for Understanding Nano-cellular Interactions. Adv. Exp. Med. Biol..

[3] Yuan X., Zhang X., Sun L., Wei Y., Wei X. (2019). Cellular Toxicity and Immunological Effects of Carbon-based Nanomaterials. Part. Fibre Toxicol..

[4] Luo B., Liu G., Wang L. (2016). Recent advances in 2D materials for photocatalysis. Nanoscale.

[5] Chen Y., Tan C., Zhang H., Wang L. (2015). Two-dimensional graphene analogues for biomedical applications. Chem. Soc. Rev..

[6] Bolibok P., Roszek K., Wiśniewski M. (2018). Graphene Oxide-Mediated Protection from Photodamage. J. Phys. Chem. Lett..

[7] Zhao C., Tan A., Pastorin G., Ho H.K. (2013). Nanomaterial scaffolds for stem cell proliferation and differentiation in tissue engineering. Biotechnol. Adv..

[8] Bhattacharya K., Mukherjee S.P., Gallud A., Burkert S.C., Bistarelli S., Bellucci S., Bottini M., Star A., Fadeel B. (2016). Biological interactions of carbon-based nanomaterials: From coronation to degradation. Nanomedicine.

[9] Shvedova A.A., Yanamala N., Kisin E.R., Tkach A.V., Murray A.R., Hubbs A., Chirila M.M., Keohavong P., Sycheva L.P., Kagan V.E. (2014). Long-term effects of carbon containing engineered nanomaterials and asbestos in the lung: one year postexposure comparisons. Am. J. Physiol. Lung Cell Mol. Physiol..

[10] Morimoto Y., Horie M., Kobayashi N., Shinohara N., Shimada M. (2013). Inhalation toxicity assessment of carbon-based nanoparticles. Acc. Chem. Res..

[11] Donaldson K., Poland C.A. (2013). Nanotoxicity: challenging the myth of nano-specific toxicity. Curr. Opin. Biotechnol..

[12] Chng E.L.K., Chua C.K., Pumera M. (2014). Graphene oxide nanoribbons exhibit significantly greater toxicity than graphene oxide nanoplatelets. Nanoscale.

[13] Madannejad R., Shoaie N., Jahanpeyma F., Darvishi M.H., Azimzadeh M., Javadi H. (2019). Toxicity of carbon-based nanomaterials: Reviewing recent reports in medical and biological systems. Chem. Biol. Interact.

[14] Tian R., Long X., Yang Z., Lu N., Peng Y. (2020). Formation of a bovine serum albumin diligand complex with rutin and single-walled carbon nanotubes for the reduction of cytotoxicity. Biophys. Chem..

[15] Casey A., Herzog E., Lyng F.M., Byrne H.J., Chambers G., Davoren M. (2008). Single walled carbon nanotubes induce indirect cytotoxicity by medium depletion in A549 lung cells. Toxicol. Lett..

[16] Olivares R., Rodil S.E., Arzate H. (2007). Osteoinduction properties of graphite-like amorphous carbon films evaluated in-vitro. Diamond Relat. Mater..

[17] Jain S., Sharma A., Basu B. (2013). In vitro cytocompatibility assessment of amorphous carbon structures using neuroblastoma and Schwann cells. J. Biomed. Mater. Res. B Appl. Biomater..

[18] Popov C., Kulisch W., Reithmaier J.P., Dostalova T., Jelinek M., Anspach N., Hammann C. (2007). Bioproperties of nanocrystalline diamond/amorphous carbon composite films. Diamond Relat. Mater..

[19] Werengowska-Ciećwierz K., Wiśniewski M., Terzyk A.P., Roszek K., Czarnecka J., Bolibok P., Rychlicki G. (2015). Conscious Changes of Carbon Nanotubes Cytotoxicity by Manipulation with Selected Nanofactors. Appl. Biochem. Biotechnol..

[20] Cagliani R., Gatto F., Bardi G. (2019). Protein Adsorption: A Feasible Method for Nanoparticle Functionalization. Materials.

[21] Baimanov D., Cai R., Chen C. (2019). Understanding the Chemical Nature of Nanoparticle-Protein Interactions. Bioconjug. Chem..

[22] Liu N., Tang M., Ding J. (2020). The interaction between nanoparticles-protein corona complex and cells and its toxic effect on cells. Chemosphere.

[23] Mohajeri M., Behnam B., Sahebkar A. (2019). Biomedical applications of carbon nanomaterials: Drug and gene delivery potentials. J. Cell Physiol..

[24] Boncel S., Kyzioł-Komosińska J., Krzyżewska I., Czupioł J. (2015). Interactions of carbon nanotubes with aqueous/aquatic media containing organic/inorganic contaminants and selected organisms of aquatic ecosystems—A review. Chemosphere.

[25] Rodriguez-Yañez Y., Muñoz B., Albores A. (2013). Mechanisms of toxicity by carbon nanotubes. Toxicol. Mech. Methods.

[26] (2010). Directive 2010/63/EU of the European Parliament and of the Council of 22 September 2010 on the protection of animals used for scientific purposes. Off. J. Eur. Union.

[27] Thurnherr T., Brandenberger C., Fischer K., Diener L., Manser P., Maeder-Althaus X., Kaiser J.P., Krug H.F., Rothen-Rutishauser B., Wick P. (2011). A comparison of acute and long-term effects of industrial multiwalled carbon nanotubes on human lung and immune cells in vitro. Toxicol. Lett..

[28] Medepalli K., Alphenaar B., Raj A., Sethu P. (2011). Evaluation of the direct and indirect response of blood leukocytes to carbon nanotubes (CNTs). Nanomedicine.

[29] Palomäki J., Karisola P., Pylkkänen L., Savolainen K., Alenius H. (2010). Engineered nanomaterials cause cytotoxicity and activation on mouse antigen presenting cells. Toxicology.

[30] Hirano S., Fujitani Y., Furuyama A., Kanno S. (2010). Uptake and cytotoxic effects of multi-walled carbon nanotubes in human bronchial epithelial cells. Toxicol. Appl. Pharmacol..

[31] Chng E.L.K., Pumera M. (2015). Toxicity of graphene related materials and transition metal dichalcogenides. RSC Adv..

[32] Demir Cakan R., Titirici M.M., Antonietti M., Cui G., Maier J., Hu Y.-S. (2008). Hydrothermal carbon spheres containing silicon nanoparticles: synthesis and lithium storage performance. Chem. Commun..

[33] Wei J., Liang Y., Zhang X., Simon G.P., Zhao D., Zhang J., Jiange S., Wang H. (2015). Controllable synthesis of mesoporous carbon nanospheres and Fe-N/carbon nanospheres as efficient oxygen reduction electrocatalysts. Nanoscale.

[34] Werengowska-Ciećwierz K., Wiśniewski M., Terzyk A.P., Gurtowska N., Olkowska J., Kloskowski T., Drewa T.A., Kiełkowska U., Drużyński S. (2014). Nanotube-mediated efficiency of cisplatin anticancer therapy. Carbon.

[35] Nowacki M., Wiśniewski M., Werengowska-Ciećwierz K., Roszek K., Czarnecka J., Łakomska I., Kloskowski T., Tyloch D., Debski R., Pietkun K. (2015). Nanovehicles as a Novel Target Strategy for Hyperthermic Intraperitoneal Chemotherapy: A Multidisciplinary Study of Peritoneal Carcinomatosis. Oncotarget.

[36] Arayachukiat S., Seemork J., Pan-In P., Amornwachirabodee K., Sangphech N., Sansureerungsikul T., Sathornsantikun K., Vilaivan C., Shigyou K., Pienpinijtham P. (2015). Bringing macromolecules into cells and evading endosomes by oxidized carbon nanoparticles. Nano Lett..

[37] Mihalchik A.L., Ding W., Porter D.W., McLoughlin C., Schwegler-Berry D., Sisler J.D., Stefaniak A.B., Snyder-Talkington B.N., Cruz-Silva R., Terrones M. (2015). Effects of nitrogen-doped multi-walled carbon nanotubes compared to pristine multi-walled carbon nanotubes on human small airway epithelial cells. Toxicology.

[38] Figarol A., Pourchez J., Boudard D., Forest V., Akono C., Tulliani J.M., Lecompte J.P., Cottier M., Bernache-Assollant D., Grosseau P. (2015). In vitro toxicity of carbon nanotubes, nano-graphite and carbon black, similar impacts of acid functionalization. Toxicol. In Vitro.

[39] Blundell E.L.C.J., Healey M.J., Holton E., Sivakumaran M., Manstana S., Platt M. (2016). Characterisation of the protein corona using tunable resistive pulse sensing: determining the change and distribution of a particle’s surface charge. Anal. Bioanal. Chem..

[40] Wick P., Manser P., Limbach L., Dettlaff-Weglikowska U., Krumeich F., Roth S., Stark W., Bruinink A. (2007). The Degree and Kind of Agglomeration Affect Carbon Nanotube Cytotoxicity. Toxicol. Lett..

[41] Prajapati S.K., Malaiya A., Kesharwani P., Soni D., Jain A. (2020). Biomedical applications and toxicities of carbon nanotubes. Drug Chem. Toxicol..

[42] Lesniak A., Fenaroli F., Monopoli M.P., Åberg C., Dawson K.A., Salvati A. (2012). Effects of the presence or absence of a protein corona on silica nanoparticle uptake and impact on cells. ACS Nano.

